# A major quantitative trait locus *qSBR11* from *Oryza sativa* ‘Milyang 44’ confers resistance to rice stink bugs

**DOI:** 10.1270/jsbbs.25084

**Published:** 2026-06-16

**Authors:** Masami Tsuzuki, Mitsuru Nakamura, Hiroyuki Kokaji, Hiroto Yamaguchi, Kazuhiko Sugiura, Tomofumi Yoshida, Shuhei Kato, Kenichiro Mori, Hiroko Ohashi, Utako Yamanouchi, Shuichi Fukuoka, Ayahiko Shomura, Koichi Toyokura, Shiro Fukuta

**Affiliations:** 1 Aichi Agricultural Research Center (AARC), 1-1 Yazako-Sagamine, Nagakute, Aichi 480-1193, Japan; 2 GRA&GREEN Inc., Furo-cho, Chikusa-ku, Nagoya, Aichi 464-8601, Japan; 3 Institute of Crop Science, National Agriculture and Food Research Organization (NARO), 2-1-1 Kannondai, Tsukuba, Ibaraki 305-8602, Japan

**Keywords:** *Oryza sativa* L., stink bug resistance, QTL mapping, marker-assisted selection, pecky rice, ‘Milyang 44’

## Abstract

Stink bugs are major insect pests that damage rice after heading, causing pecky rice and sterility, which significantly reduce grain quality and yield. We identified a major quantitative trait locus (QTL) for stink bug resistance derived from the resistant donor variety ‘Milyang 44’ and explored DNA markers useful for marker-assisted selection in rice breeding. QTL analysis using an F_5_ population (n = 94) identified a major QTL, *qSBR11*, on chromosome 11, which explained 28.46% of phenotypic variance in Pecky Rice Index. Fine mapping of the BC_1_F_3_ and BC_5_F_3_ populations narrowed the candidate region of *qSBR11* to approximately 616 kbp. In the validation experiments, individuals selected using the marker C5_indel9205 located within this candidate region showed significantly reduced pecky rice rate. Additionally, the marker genotype was highly consistent with phenotypes of 22 tested varieties, and genomic analysis of 685 accessions from TASUKE+ in the Rice Annotation Project Database showed that the resistant allele was rare in *japonica* cultivars. Therefore, that *qSBR11* is a promising target for breeding *japonica* rice. This study identified a major QTL for stink bug-induced pecky rice resistance and demonstrated the utility of a reliable DNA marker for practical breeding.

## Introduction

Rice (*Oryza sativa* L.) is a staple crop that serves as a food source for approximately half of the world’s population, and global food security is directly linked to its sustainable production (Mohidem *et al.* 2022). During rice cultivation, various biotic stressors affect both yield and quality. Stink bugs are major insect pests that cause damage after heading. Stink bug infestation induces spikelet sterility and kernel discoloration (pecky rice), which is characterized by brown spots on the surface of brown rice, leading to reduced grain quality and yield. Stink bug-induced damage is a serious problem affecting sustainable production in rice-growing regions worldwide, including Japan (Bhavanam *et al.* 2021, Bhavanam and Stout 2022, Krinski and Foerster 2017, Lee *et al.* 1993, Swanson and Newsom 1962, Tindall *et al.* 2005). Recently, the expansion of abandoned and fallow fields has increased the prevalence of gramineous weeds that serve as sources of stink bug infestation, and climate warming has further exacerbated this problem by increasing the number of generations and overwintering survival (Ito 2004, Kiritani 2007, Yamashita 2008). Although chemical insecticides are the primary control measures, concerns regarding their environmental impact and emergence of insecticide-resistant pests highlight the need for sustainable pest-management strategies. Therefore, introducing genetically resistant crop cultivars is an effective strategy for sustainable rice cultivation.

Rice cultivars differ in their response to stink bug damage (Bhavanam and Stout 2022, Jahn *et al.* 2004, Sugiura *et al.* 2022). For instance, the *indica* cultivar ‘Milyang 44’ exhibits strong resistance to stink bugs, *Leptocorisa chinensis* and *Cletus punctiger* (Nakamura *et al.* 2017, Sugiura *et al.* 2017). Nakamura *et al.* (2017) demonstrated through field trials that the pecky rice rate was significantly lower in ‘Milyang 44’ (18–47%) than in the susceptible cultivar ‘Aichinokaori SBL’. Pecky rice is produced when the kernels damaged by stink bugs during feeding are invaded by bacteria and fungi that proliferate and cause grain browning (Hollay *et al.* 1987, Marchetti and Petersen 1984). ‘Milyang 44’ is characterized by thickened husk cell walls containing lignin, which is considered to structurally impede feeding by stink bugs (Nakamura *et al.* 2020). The development of cultivars with these resistance traits is necessary to improve crop growth and yield (Sugiura and Nakamura 2023). However, phenotypic evaluation of stink bug resistance is extremely labor-intensive and unsuitable for large-scale selection of breeding lines, limiting the development of resistant cultivars. Marker-assisted selection based on resistance-related quantitative trait loci (QTLs) is necessary to efficiently introduce resistance in other cultivars. To date, no QTL for stink bug-induced pecky rice resistance has been identified (Sugiura *et al.* 2017). Therefore, in this study, we identified the major QTLs for stink bug resistance in ‘Milyang 44’ and evaluated their effectiveness in marker-assisted selection. Additionally, we analyzed the applicability of this QTL (*qSBR11*) to other cultivars and its distribution across cultivar groups to provide insights into its practical use in breeding programs.

## Materials and Methods

### Plant materials

The plant materials used in this study are summarized in Fig. 1 and Table 1. To evaluate the effects of stink bug resistance QTLs on a *japonica* genetic background, the *japonica* line ‘Chubu 129’ was crossed with the *indica* cultivar ‘Milyang 44’, and a resistant intermediate breeding line, ‘Ikame 1470’, which showed stable resistance, was developed. QTL analysis was conducted using a population derived from a cross between ‘Ikame 1470’ and the *japonica* cultivar ‘Natsukirari’ (Fig. 1A), consisting of 33 F_5_ lines from which two or three plants per line were selected, resulting in a total of 94 plants for evaluation. As residual heterozygosity is expected in F_5_ populations, individual plants were treated as independent replicates for QTL analysis rather than aggregated by line. The resistant F_5_ line was advanced to the F_6_ generation and designated ‘Mi7’, which was subsequently used for fine mapping and validation of marker effects. This study was conducted as part of a practical rice breeding program aimed at developing stink bug-resistant cultivars for cultivation in Japan. Multiple populations with different genetic backgrounds were generated to facilitate genetic analysis and cultivar development. BC_1_F_3_ populations derived from the cross between ‘Natsukirari’ and ‘Mi7’ were used for initial fine mapping (Fig. 1B). Meanwhile, the BC_5_F_3_ populations with more extensive backcrossing to ‘Aichinokokoro’ were employed to validate the QTL effect in a genetic background approaching that of an elite *japonica* cultivar and to assess its applicability for practical breeding (Fig. 1C). Additionally, an F_2_ population derived from the cross between ‘Natsukirari’ and ‘Mi7’ was used to validate the marker effects (Fig. 1D).

### Evaluation of resistance phenotypes

Resistance was evaluated as described by Nakamura *et al.* (2017) and Sugiura *et al.* (2017). Stink bugs (*L. chinensis*) were collected from weedy fields at the Aichi Agricultural Research Center and reared in net cages in a glasshouse with rice plants as a food source under natural conditions without the controlled synchronization of developmental stage or sex ratio. Based on the stable resistance of ‘Milyang 44’ at 20 days after heading (Nakamura *et al.* 2017), all plants were subjected to resistance tests at this timing. For each plant, two synchronously heading panicles were selected and all others were removed. Plants grown in 3.5-L pots were placed inside net cages (300 × 240 × 160 cm) with approximately four insects per panicle; when populations exceeded this density, additional rice plants were introduced to maintain the target. The number of test plants per cage varied by trial scale, but per-panicle insect density was consistently maintained. Within cages, replicates of the same line were spatially separated and edge-positioned plants were distributed equally across genotypes to minimize positional effects. Plants were exposed to stink bugs for 7 days in the glasshouse (ventilation at 28°C), then insects were eliminated by spraying with dinotefuran (Starkle Water Dispersible Granule, Hokko Chemical Industry Co., Ltd.), and plants were grown to maturity in a separate glasshouse to prevent further damage.

Resistance was evaluated post-harvest by scoring the pecky rice rate as the primary phenotype. Trained evaluators manually removed unfilled grains (empty or immature) and hull-cracked grains (premature hull cracking, which increases susceptibility to feeding) prior to evaluation. After this removal step, the remaining filled grains were defined as examined grains and were visually inspected to count undamaged grains (no visible browning or discoloration). Pecky grains (browning from stink bug feeding) were derived as the difference between examined and undamaged grains, rather than counted directly, because they often break during processing. Under our controlled conditions, non-stink bug grain defects (e.g., from disease or abiotic stress) were negligible. The pecky rice rate was calculated as:


$$\text{Pecky rice rate}=\;\frac{\left(\text{Number of examined grains}-\text{Number of undamaged grains}\right)}{\text{Number of examined grains}}$$

where:


Number of examined grains=Total grains−(Unfilled grains+Hull-cracked grains)

To normalize environmental variation among tests, the Pecky Rice Index (PRI) was calculated, as stink bug damage severity is influenced by insect developmental stage and density (Bhavanam *et al.* 2021). In each test, a susceptible control was included, and PRI was obtained by normalizing the pecky rice rate of each sample to that of the control.


$$\text{PRI}=\frac{\text{Pecky rice rate}}{\text{Pecky rice rate of the susceptible control}}$$

### QTL analysis

Overall, 94 F_5_ plants derived from a cross between the susceptible cultivar ‘Natsukirari’ and resistant line ‘Ikame 1470’, which carries resistance from ‘Milyang 44’, were used for QTL analysis. Genomic DNA was extracted from leaf tissues using the protocol described by Monna *et al.* (2002). Single nucleotide polymorphism (SNP) genotyping assays were performed using the 96.96 Dynamic Array IFC chip following the “SNP type 96 × 96 v1” protocol provided by Standard BioTools Inc. From the obtained genotype data, markers were selected based on polymorphisms between ‘Ikame 1470’ and ‘Chubu 129’. Since ‘Ikame 1470’ was developed through backcrossing to ‘Chubu 129’ (Fig. 1A), these markers are expected to be enriched for ‘Milyang 44’-derived genomic regions where resistance loci are likely to reside. To enable targeted detection of resistance loci derived from ‘Milyang 44’, markers were intentionally prioritized in genomic regions expected to contain introgressed segments rather than for uniform genome-wide coverage. These pre-selected markers were confirmed to also show polymorphisms between the mapping parents (‘Natsukirari’ and ‘Ikame 1470’). Markers with complete genotype data for all individuals were retained, resulting in a final set of 85 markers for QTL analysis (Supplemental Table 1). All physical positions reported in this study are based on the rice reference genome assembly IRGSP-1.0 (Kawahara *et al.* 2013). Genetic distances were estimated using the est.map function in R/qtl (v1.70) (Broman *et al.* 2003) with the option map.function = “morgan”. Composite interval mapping based on Haley–Knott regression was also performed. Phenotypic evaluations of the mapped population were performed in four independent trials between September and October 2018. In each trial, a susceptible cultivar was tested simultaneously as a control to calculate the PRI, which was used as the phenotypic value. For QTL detection, a random permutation test was conducted 1,000 times, and a 5% significance threshold was used as the logarithm of the odds (LOD) score cutoff.

As a complementary analysis, a linear model was fitted using raw pecky rice rate with trial included as a covariate. For each marker, the model Pecky rice rate ~ Genotype + Test was fitted, where genotype represents the marker genotype (A, B, or H) and test represents the experimental batch. The significance of the genotype effect was evaluated by analysis of variance (ANOVA) through comparison of the full model with a reduced model containing only the trial effect. LOD scores were calculated as –log_10_(*p*-value) derived from the ANOVA F-test. This analysis was performed in R version 4.5.2 using the lm() function.

### Fine mapping analysis

For QTL identification, BC_1_F_3_ lines derived from a cross between ‘Natsukirari’ and ‘Mi7’ were used for mapping analysis. InDel markers (Yonemaru *et al.* 2015) and SSR markers (McCouch *et al.* 2002) were employed to examine the genotypes of each individual within the candidate QTL region, and selected lines were assigned IDs beginning with “QTLM”. For each line, seven plants were evaluated as replicates to assess stink bug resistance. Resistance evaluations were conducted twice in 2023 according to the heading dates of the tested lines. In both trials, the susceptible cultivar ‘Natsukirari’ was simultaneously exposed to stink bugs and used as the susceptible control cultivar.

To further narrow down the detected QTL candidate region, BC_5_F_3_ lines derived from a cross between ‘Aichinokokoro’ and ‘Mi7’ were analyzed. DNA markers were used to genotype the chromosomal region containing the QTL, and recombinant individuals within this region were selected for resistance evaluation. For each line, six plants were evaluated as replicates. Resistance assays were performed twice in 2024. ‘Aichinokokoro’ was used as a susceptible control cultivar in the first test; however, no ‘Aichinokokoro’ plants at the appropriate heading stage were observed in the second test. Therefore, a BC_5_F_3_ line that was fixed for the susceptible genotype across the entire candidate QTL region (C5_indel9182–MT099100, approximately 1.36 Mbp) was selected as the susceptible control line, and the PRI was calculated.

Lines were classified as resistant (R) if their PRI values were significantly lower than the susceptible control (Dunnett’s test, *p* < 0.05), and as susceptible (S) if no significant difference was detected.

### Whole-genome resequencing (WGS) and marker development

Among the BC_1_F_3_ population used for fine mapping, four lines (‘QTLM1’, ‘QTLM3’, ‘QTLM4’, and ‘QTLM6’) were selected based on phenotypic evaluations and subjected to WGS. Genomic DNA was extracted from 15 seeds and ground into powder using a DNeasy Plant Mini Kit (QIAGEN K.K., Hilden, Germany). WGS, including library preparation, was outsourced to Genome-Lead Co., Ltd. (Kagawa, Japan). Libraries were constructed using the MGIEasy FS DNA Library Prep Set (MGI Tech Co., Ltd., Shenzhen, China) and sequenced on the DNBSEQ-G400RS platform in the 150 bp paired-end mode. Raw reads were quality-filtered using FastQC (Andrews 2010) and mapped to the *O. sativa* reference genome assembly IRGSP-1.0 (Kawahara *et al.* 2013) using BWA-MEM (https://arxiv.org/abs/1303.3997) with default settings. Thereafter, the aligned sequences were visually inspected using IGV (Robinson *et al.* 2011). Based on the polymorphisms within the candidate QTL region, PCR-based DNA markers (designated as MT numbers) were developed and used in subsequent analyses.Validation of DNA marker effects.

To validate the marker effect in an independent segregating population, an F_2_ population was developed from a cross between the resistant line ‘Mi7’ and susceptible cultivar ‘Natsukirari’. The genotype of each individual was determined using the C5_indel9205 marker. Individuals were classified as homozygous resistant (RR; R-type, 136 bp fragment) or homozygous susceptible (SS; S-type, 119 bp fragment). Heterozygous individuals were identified but excluded from phenotypic evaluation to focus on fixed genotypes relevant to breeding.

Resistance evaluations were conducted twice in 2024 at the heading stage. In the first test, 14 individuals (eight R-type and six S-type) were evaluated, and in the second test, nine individuals (three R-type and six S-type) were evaluated. Differences in pecky rice rate between genotypes were analyzed using the Wilcoxon rank-sum test, and 95% confidence intervals (CIs) for the location shift (R-type – S-type) were calculated.

The WGS data used in this study are available in the DDBJ database under BioProject accession number PRJDB35668.

### Genotyping of diverse varieties

Sugiura *et al.* (2017) evaluated stink bug resistance in 503 rice lines originating from diverse regions, including Asia, the Americas, Europe, and Oceania, between 2008 and 2014. Of these, 22 lines with reliably assessed resistance over several years were genotyped using the DNA marker C5_indel9205.

## Results

### QTL analysis

In this study, an F_5_ population (94 individuals) derived from a cross between the susceptible cultivar ‘Natsukirari’ and resistant line ‘Ikame 1470’ was used for QTL analysis. Genotype analysis revealed that 79 of 85 markers (93%) segregated within the population, with heterozygous individuals observed across multiple lines (Supplemental Table 1), indicating that genetic fixation was not yet complete at the F_5_ generation. Therefore, individual plants were treated as independent genetic entries for QTL analysis. Supplemental Table 2 presents the results of the resistance assay for ‘Ikame 1470’. Each individual was evaluated for resistance using four stink bug infestation tests conducted according to the heading date of the panicles. The pecky rice rate in this population ranged from 16.9 to 81.2%. In each test, the pecky rice rate in the susceptible control cultivar was stable, ranging from 59.2 to 60.8%. Accordingly, this value was used as the baseline (1.0) to normalize the pecky rice rate of each plant, and the PRI was calculated. The PRI of the F_5_ population exhibited a continuous distribution (Fig. 2). QTL analysis was conducted using the Haley–Knott regression method with PRI as the phenotypic value. A major QTL was detected between SNP markers FA1577 (14.3 Mbp) and FA1617 (21.8 Mbp) on chromosome 11 (designated as *qSBR11*). The peak LOD score of *qSBR11* was 6.83 with a *p*-value of 2.42 × 10^–7^, explaining 28.46% of the phenotypic variance in PRI (Table 2, Supplemental Fig. 1).

To assess whether QTL detection was influenced by PRI normalization, a validation analysis was conducted using raw pecky rice rate with trial included as a covariate in a linear model. The distribution of raw pecky rice rate was consistent across the four trials, with median values ranging from 41.8 to 47.5% and a coefficient of variation of trial medians of 5.23% (Supplemental Fig. 2). No significant differences were detected among trials (Kruskal–Wallis test, *p* = 0.77). Linear model analysis confirmed the presence of a major QTL on chromosome 11 at a similar position (peak at 17.95 Mb, LOD = 6.38, *p* = 4.19 × 10^–7^), indicating that *qSBR11* was consistently detected regardless of the normalization method (Supplemental Fig. 3).

### Fine mapping

For fine mapping, BC_1_F_3_ and BC_5_F_3_ populations were developed using ‘Mi7’, a selfed progeny line selected for stink bug resistance from the F_5_ population as the resistant parent. Supplemental Table 2 presents the results of the resistance assay for the ‘Mi7’ cultivar.

Among the BC_1_F_3_ population, resistant lines ‘QTLM1’ and ‘QTLM3’ and susceptible lines ‘QTLM4’ and ‘QTLM6’ were selected for WGS. Based on the sequence information obtained, new DNA markers (designated as MT numbers, Supplemental Table 3) were designed and used to genotype the candidate QTL regions. Recombinant lines within the *qSBR11* region were identified in the BC_1_F_3_ population, and their resistance phenotypes were re-evaluated. In ‘QTLM2’, the upstream region of the marker C5_indel9205 was not genetically fixed, and individuals with different genotypes were present within the line. Therefore, individuals carrying the R-type allele were classified as ‘QTLM2(R)’, whereas those carrying the S-type allele were classified as ‘QTLM2(S)’. Further analysis of both groups showed that ‘QTLM1’, ‘QTLM2(R)’, and ‘QTLM3’ had significantly lower PRI values than the susceptible control, ‘Natsukirari’. By contrast, the PRI values of ‘QTLM2(S)’, ‘QTLM4’, and ‘QTLM6’ were not significantly different from that of the control. Overall, these findings localized *qSBR11* between markers C5_indel9182 and MT099100 (Fig. 3A).

To further refine the position of *qSBR11*, the BC_5_F_3_ population was analyzed. ‘Aichinokokoro’ was used as the susceptible control in the first test; however, no plants of this cultivar reached the heading stage at the appropriate time in the second test. Therefore, the line ‘R6Mi18’, which was fixed for the susceptible genotype between C5_indel9182 and MT099100, was used as an internal susceptible control. Notably, no significant difference (Welch’s *t*-test, *p* = 0.268) was observed in the pecky rice rate between ‘R6Mi18’ (65.7%) and ‘Aichinokokoro’ (74.3%). Similar to ‘Natsukirari’, ‘Aichinokokoro’ carried an S-type allele (*qSBR11*-S) in this interval. In this study, ‘R6Mi7’ and ‘R6Mi12’ exhibited significantly lower PRI values than the susceptible controls, with no significant differences between ‘R6Mi15’ and ‘R6Mi17’. Consequently, the *qSBR11* locus was delimited to a 616 kbp region between MT212213 and MT227228 (Fig. 3B).

### Validation of the DNA marker

In the F_2_ population, individuals were genotyped using the C5_indel9205 marker, and only homozygous plants (R-type and S-type) were analyzed for pecky rice rate. In the first test (n = 8 for R-type, n = 6 for S-type), the pecky rice rate was significantly lower in R-type than in S-type (Wilcoxon rank-sum test, W = 0, *p* = 0.0024). The estimated location shift was –30.5 (95% CI: –40.0 to –15.0).

Similarly, the second test (n = 3 for R-type, n = 6 for S-type) confirmed a significantly lower pecky rice rate in R-type (W = 0, *p* = 0.0238), with an estimated location shift of –31.0 (95% CI: –46.0 to –15.0). Despite the limited sample size, these consistent results across two independent evaluations support the association between the C5_indel9205 genotype and pecky rice rate (Fig. 4).

### Genotyping of qSBR11 in diverse varieties

Table 3 presents the PRI values for the 22 accessions evaluated between 2008 and 2011 along with their resistance classifications and genotypes for the C5_indel9205 marker. Resistance was evaluated annually over several years, and varieties with consistently low pecky rice rate were classified as resistant. The pecky rice rate for each variety in 2008 was reported by Sugiura *et al.* (2017).

Among the 14 varieties classified as resistant, 13 (excluding ‘Dellmont’) carried the R genotype in the *qSBR11* region. In contrast, all eight varieties classified as susceptible, as well as the three susceptible control cultivars, carried the S genotype.

## Discussion

Stink bugs are serious pests in rice cultivation globally. Although chemical methods have traditionally been employed for pest control, the development of resistant varieties is a sustainable strategy with no environmental impact. Sugiura *et al.* (2017) identified ‘Milyang 44’ as a resistant variety to stink bugs, and Nakamura *et al.* (2020) reported that this resistance was attributable to structural traits that prevented feeding, specifically the thickening of the lignin-containing cell wall in the hull. In this study, we identified a QTL for stink bug resistance in ‘Milyang 44’, delimit its location through fine mapping, and evaluate its applicability for breeding.

In the present study, a small population of 94 plants was developed using the intermediate line ‘Ikame 1470’, which carries resistance from ‘Milyang 44’. QTL analysis was performed based on relatively low-density genotype data obtained from 85 SNP markers. A major QTL (*qSBR11*) that explained approximately 28% of the phenotypic variance was identified on chromosome 11 (Table 2, Supplemental Fig. 1). Fine mapping of this QTL was performed using BC_1_F_3_ and BC_5_F_3_ populations derived from different susceptible cultivars (‘Natsukirari’ and ‘Aichinokokoro’), narrowing the candidate region to 616 kbp (Fig. 3). The BC_5_F_3_ population could be regarded as having nearly isogenic lines, and the effects of *qSBR11* in this population strongly supported the presence and effectiveness of this locus. Furthermore, validation using the F_2_ population confirmed that the introduction of *qSBR11* reduced pecky rice rate by approximately 30% (Fig. 4), thereby demonstrating its role. Notably, the effect of *qSBR11* was consistently detected across multiple populations with different susceptible parents (Fig. 3), suggesting that this QTL may function as a resistance locus, even in diverse *japonica* backgrounds. As part of a practical breeding program, this multi-population strategy enabled simultaneous genetic analysis and cultivar development, successfully demonstrating the QTL’s robustness and wide applicability for marker-assisted selection. However, ‘Milyang 44’ tended to show stronger resistance than the BC_1_F_3_ lines (Fig. 3), ‘Ikame 1470’, and ‘Mi7’ (Supplemental Table 2), implying the presence of additional resistance factors beyond *qSBR11*. Analysis of unpublished WGS data for ‘Ikame 1470’ revealed extensive replacement by the susceptible parent genotype across multiple chromosomal regions, including chromosome 3, with a few regions retained from ‘Milyang 44’. This may have contributed to the reduced marker density and biased marker distribution observed in the QTL analysis. Future studies using populations directly derived from ‘Milyang 44’ and high-density markers may allow the identification of additional resistance loci, including potential minor QTLs, besides *qSBR11*.

Nakamura *et al.* (2020) demonstrated that ‘Milyang 44’ exhibits thickened hull cell walls with lignin-containing secondary walls, providing a structural barrier against stink bug feeding. The *qSBR11* candidate region contains 65 annotated transcripts (Rice Annotation Project Database; RAP-DB ver. 20231214; Sakai *et al.* 2013), among which cytochrome P450 and 2OG-Fe(II) oxygenase gene families are of particular interest given their established roles in secondary metabolism and cell wall modification (Fang *et al.* 2012, Pandian *et al.* 2020). Notably, *RL14*, encoding a 2OG-Fe(II) oxygenase expressed in sclerenchyma cells, has been shown to alter lignin and cellulose content, thereby affecting cell wall structure (Fang *et al.* 2012). While these functional characteristics make such genes plausible candidates underlying the observed phenotype, direct evidence linking specific genes to *qSBR11*-mediated resistance remains to be established. Future studies employing gene expression profiling in resistant versus susceptible genotypes, comparative analysis of allelic variation within the candidate region, and targeted gene editing approaches will be essential to identify the causal gene(s) and elucidate the molecular basis of *qSBR11*.

Stink bug resistance has been confirmed in several crop varieties. ‘IR64’ and PSBRc20 have been reported to be resistant to *Leptocorisa oratorius*, which also feeds on rice spikelets and causes pecky rice (Jahn *et al.* 2004). Bhavanam and Stout (2022) showed that *Oebalus pugnax* had a reduced preference for ‘Kaybonnet’, which was partly attributed to the volatile compounds emitted from the panicles. Sugiura *et al.* (2017) previously evaluated 503 varieties and lines for stink bug resistance and identified ‘Milyang 44’ and ‘CRR-99-95W’ as resistant donor parents. In the present study, the genotypes at marker C5_indel9205, located within the *qSBR11* candidate region, were examined for the 22 varieties evaluated by Sugiura *et al.* (2017). Among the 14 resistant varieties, 13 (excluding ‘Dellmont’) carried the same genotype as ‘Milyang 44’. Moreover, genotyping at markers flanking C5_indel9205 showed that most resistant varieties carried the same genotype as ‘Milyang 44’ across the *qSBR11* region (Supplemental Table 4), suggesting that the same QTL may also be functional in these varieties. An examination of 685 varieties registered in TASUKE+ for RAP-DB confirmed that ‘IR64’, which was previously reported to be resistant, carried the resistance allele at C5_indel9205. In this dataset, the resistant allele was predominantly found in *indica* cultivars and wild rice accessions, whereas most temperate *japonica* cultivars, including several elite Japanese cultivars, carried the susceptible allele (Supplemental Fig. 4). Therefore, the *qSBR11* candidate region shows strong genetic differentiation among varieties, suggesting that this pattern may be related to a specific loss of function in *japonica* or selective pressure during adaptation to temperate conditions. Notably, the *HAN1* gene (Os11t0483000-01), which contributes to adaptation to temperate climates (Mao *et al.* 2019), is located within the *qSBR11* candidate region, raising the possibility that resistance is lost during the adaptation process, warranting future investigation. The exceptional case of ‘Dellmont’, which showed resistance despite carrying the susceptible allele at *qSBR11*, suggests the involvement of alternative resistance mechanisms independent of *qSBR11*. This inconsistency may reflect different physiological pathways such as those mediated by volatile compounds, as reported in other stink bug–rice interactions (Bhavanam and Stout 2022), and further genetic analysis using ‘Dellmont’ could help elucidate these alternative resistance loci.

Considering that stink bug damage is influenced by diverse factors, including plant and insect developmental stages and insect density (Patel *et al.* 2006), accurate evaluation requires substantial labor and expertise. Consequently, the introduction of resistance in elite cultivars has been limited. In this study, we demonstrated that *qSBR11*-mediated resistance could be reliably selected using the DNA marker C5_indel9205, developed by Yonemaru *et al.* (2015). C5_indel9205 is a PCR gel-based DNA marker that can be conveniently used in future breeding programs. To our knowledge, this study represents the first genetic mapping of a major QTL for stink bug-induced pecky rice resistance using controlled infestation and provides a PCR-based DNA marker suitable for practical breeding selection, thereby establishing an important foundation for future rice breeding programs.

## Author Contribution Statement

M.N. and K.S. conceived the study. All authors contributed to the experimental design and investigation. M.N., H.Y., K.S., and T.Y. performed phenotypic evaluations. M.T., S.K., K.M., H.O., U.Y., S.F. (Fukuoka), and A.S. generated and curated the genotypic data. M.T., H.K., S.K., and K.T. performed statistical analyses. S.F. (Fukuta) managed the project and secured funding. M.T. wrote the first draft and revised the manuscript with input from all authors.

## Supplementary Material

Supplemental Figures

Supplemental Tables

## Figures and Tables

**Fig. 1.**
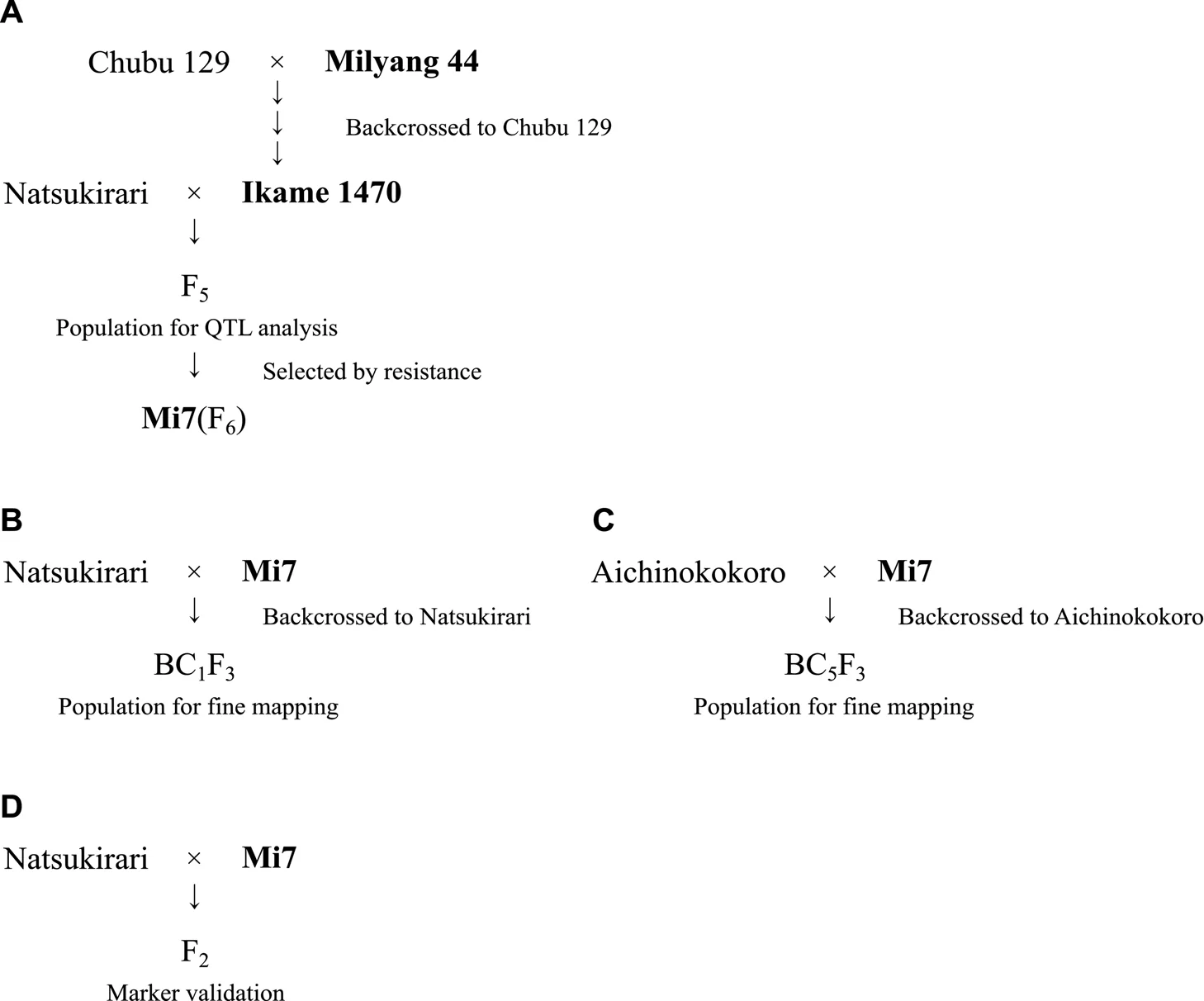
Cross design and population development. (A–D) Breeding schemes used to develop the mapping populations for quantitative trait locus (QTL), fine mapping, and marker validation as listed in Table 1. Resistant strains are indicated in bold.

**Fig. 2.**
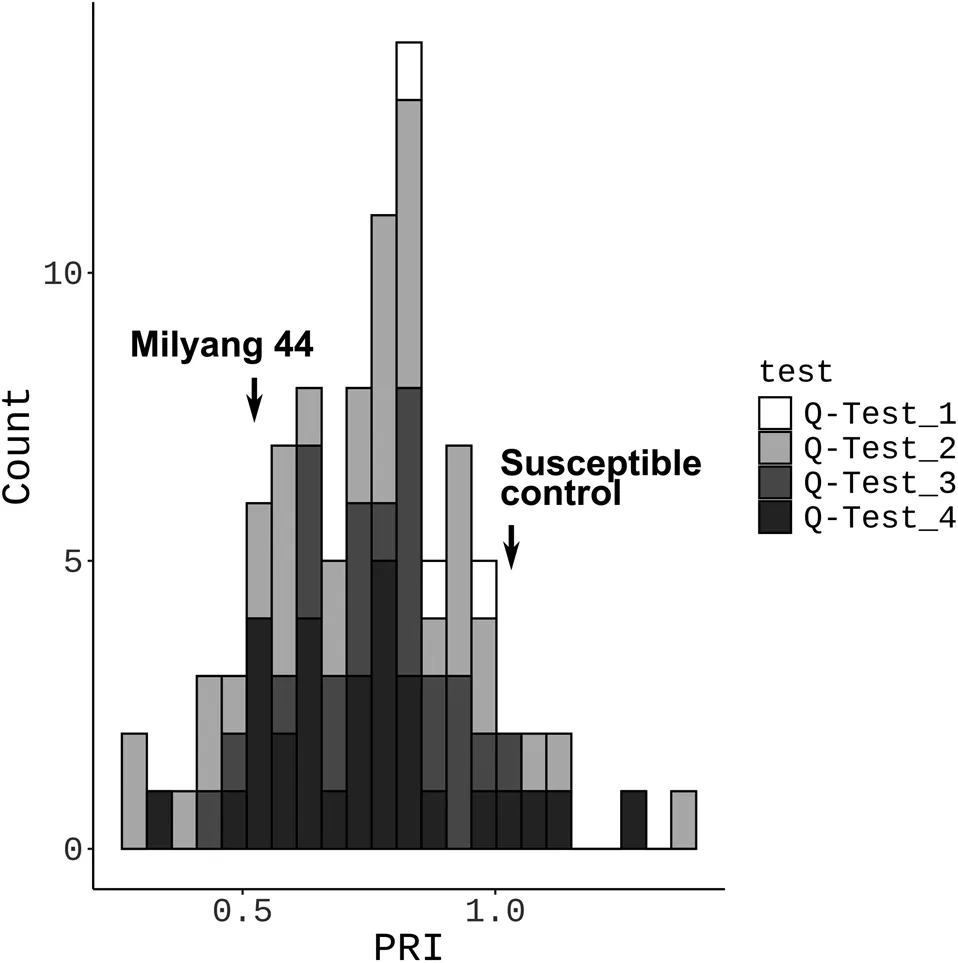
Frequency distribution of Pecky Rice Index (PRI) in F_5_ populations derived from a cross between ‘Ikame 1470’ and ‘Natsukirari’. This histogram shows the distribution of PRI values in the F_5_ population, based on four independent tests, each represented by a different color. The PRI was normalized using the susceptible control as a reference, and its value was therefore set to 1. Arrows indicate the PRI values of the susceptible control and resistant donor ‘Milyang 44’, the latter of which was evaluated using Q-Test_2.

**Fig. 3.**
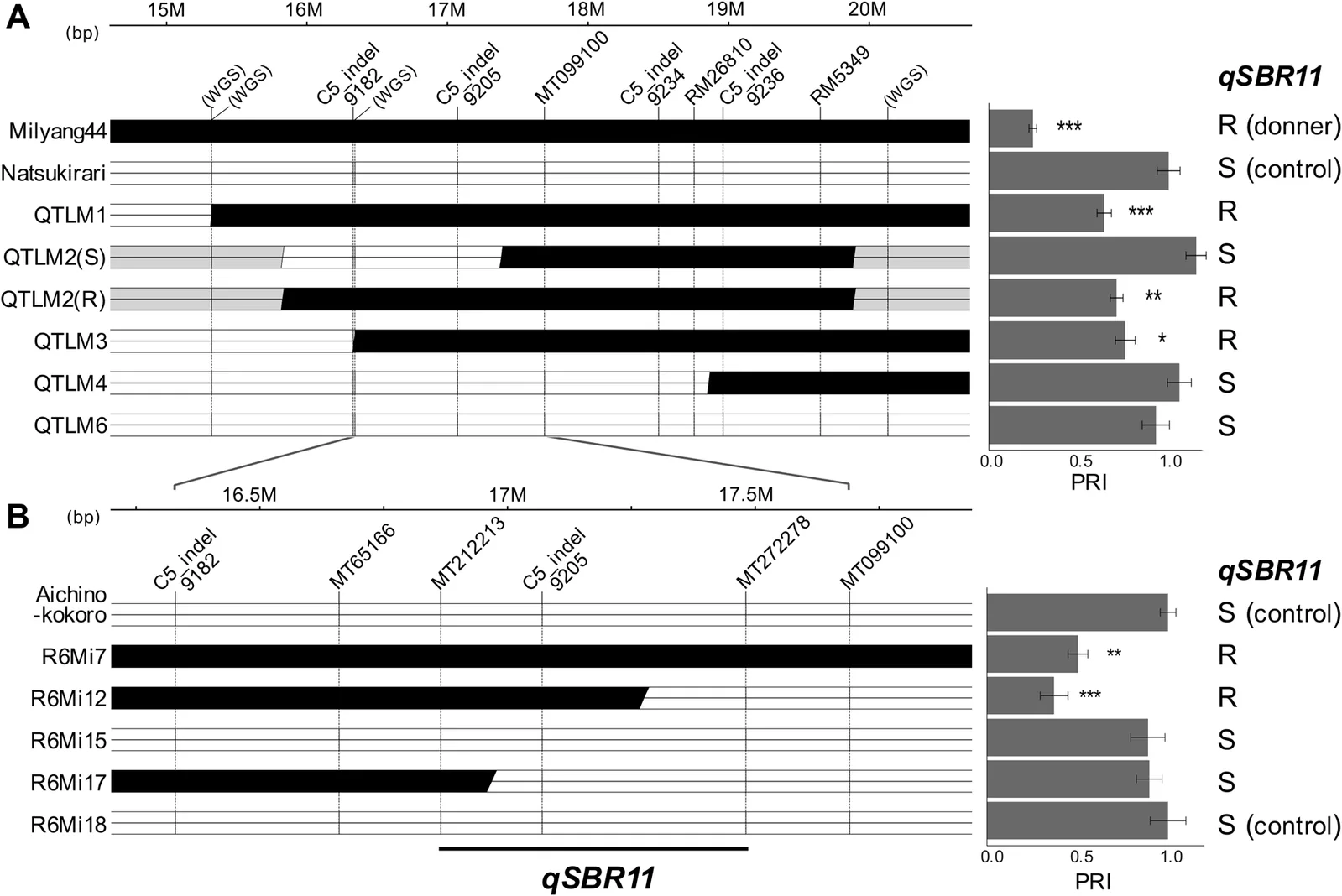
Fine mapping of *qSBR11* based on graphical genotypes and Pecky Rice Index (PRI). (A) Fine mapping using selected BC_1_F_3_ lines. The left panel shows graphical genotypes in the *qSBR11* region, with black indicating resistant R-type alleles, white indicating susceptible S-type alleles, and gray indicating undetermined regions due to missing or unsequenced data. Genotypes without marker names were inferred from whole-genome resequencing and are labeled as WGS. The middle panel shows mean PRI values for each line (n = 7), with error bars representing standard errors (SE). Asterisks denote statistical significance compared with that of the susceptible control based on one-way ANOVA followed by Dunnett’s post-hoc test: *p < 0.05, **p < 0.01, ***p < 0.001. The rightmost column indicates the inferred QTL genotype (R or S). (B) Fine mapping using BC_5_F_3_ lines. Graphical genotypes and PRI plots are presented in the same format as in panel A (n = 6 per line). Based on differential PRI values between lines with recombination in the target region, the candidate interval for *qSBR11* was refined to a 616 kbp region between MT212213 and MT227228. Graphical genotype plots were generated using GenoSee (Hashimoto 2024).

**Fig. 4.**
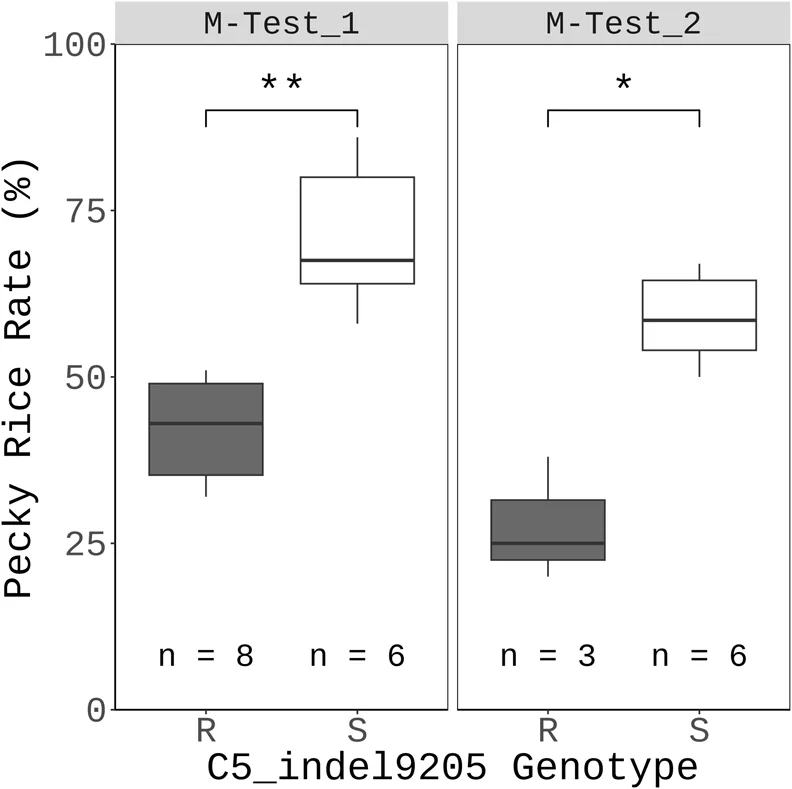
Validation of the *qSBR11* effect using the C5_indel9205 marker in an F_2_ population. Boxplots show the pecky rice rate (%) in F_2_ individuals grouped using the genotype of marker C5_indel9205, located within the *qSBR11* region. Two independent inoculation tests were conducted in 2024. Asterisks indicate significant differences between R- and S-type genotypes based on Wilcoxon rank-sum tests: *p < 0.05, **p < 0.01. Sample sizes (n) are indicated in the figure.

**Table 1.** Parental combinations, generations, and purposes of mapping populations used in this study (Visualized in Fig. 1)

Name	Parental combination	Generation	Purpose of use	Notes
Ikame 1470	Chubu 129 × Milyang 44	BC_1_F_7_	Intermediate line for QTL introgression	Used as resistant parent in mapping population
F_5_ population	Natsukirari × Ikame 1470	F_5_	QTL mapping	94 individuals used for primary QTL analysis
Mi7	Selected from F_5_ (resistant phenotype)	F_6_	Material for fine mapping & validation	Progeny of resistant individual from F_5_
BC_1_F_3_ population	Natsukirari × Mi7	BC_1_F_3_	Fine mapping	Low backcross generation
BC_5_F_3_ population	Aichinokokoro × Mi7	BC_5_F_3_	Fine mapping	Advanced backcross with high genetic background recovery
F_2_ validation population	Natsukirari × Mi7	F_2_	Marker validation	Used to evaluate genotype–phenotype association

**Table 2.** QTL identified for stink bug resistance in the F_5_ population

QTL name	Chr	Position (cM)	Marker interval	LOD	Explained variance (%)	*p*-value (F-test)	Additive effect
*qSBR11*	11	30.0	FA1577–FA1617	6.83	28.46	2.42 × 10^–7^	–0.113

Phenotypic trait: Pecky Rice Index (PRI). Additive effect estimates are based on Haley–Knott regression and represent the effect of the resistant allele (B); negative values indicate reduced PRI (increased resistance).

**Table 3.** Genotypic and phenotypic evaluation of *qSBR11* in diverse cultivars. Genotypic and phenotypic data of 22 cultivars evaluated for pecky rice resistance between 2008 and 2011. R = resistant, S = susceptible. Dashes (—) indicate that data were not available for that year

Cultivar	Origin	C5_indel9205 Genotype	Resistance	PRI			
				2008*^a^*	2009	2010	2011
Guangluai 4	China	R	R	0.146	0.012	0.300	0.298
Nanjing 11	China	R	R	0.250	0.091	0.424	0.215
GP242	China	R	R	—	0.075	0.383	0.212
Yanxuan 203	China	R	R	—	0.052	0.212	0.179
Kenanxuan 10	China	R	R	0.211	0.106	0.282	0.251
GP206	China	R	R	—	0.151	0.340	0.173
Ukoku	China Taiwan (landrace)	R	R	0.440	0.071	0.053	0.222
TI-11-8		R	R	—	0.072	0.147	0.224
IR 56	IRRI	R	R	0.204	0.066	0.805	0.485
Zhaiyeqing 8	China	R	R	0.205	0.135	0.251	0.218
Yanxuan	China	R	R	—	0.036	0.432	0.178
GP201	China	R	R	—	0.118	0.091	0.491
Yangdao 1	China	R	R	—	0.127	0.142	0.203
Dellmont	USA	S	R	—	0.104	0.735	0.328
Shuangfeng 4	China	S	S	—	2.261	1.182	1.021
Yangnuo 5	China	S	S	—	0.868	—	—
Nankeng 11	China	S	S	—	2.175	—	—
TIMIS		S	S	2.000	—	1.188	1.054
Dian Yu 1		S	S	2.833	—	0.843	0.825
Calrose No.2	USA	S	S	1.667	—	—	—
Jakou	Japan (landrace)	S	S	2.333	—	—	—
Henroyori	Japan (landrace)	S	S	—	—	0.907	1.023

^*a*^ Data from 2008 were previously reported in Sugiura *et al.* (2017).
