# Supplementary material for: A major quantitative trait locus qSBR11 from Oryza sativa ‘Milyang 44’ confers resistance to rice stink bugs
Source: Breed Sci. 2026 Jun 16;76(3):275–84. doi: 10.1270/jsbbs.25084 (PMC13709644; doi:10.1270/jsbbs.25084)
Supplement: Supplementary file 1 [file 76_275_s1.pdf]

doi: 10.1270/jsbbs.25084

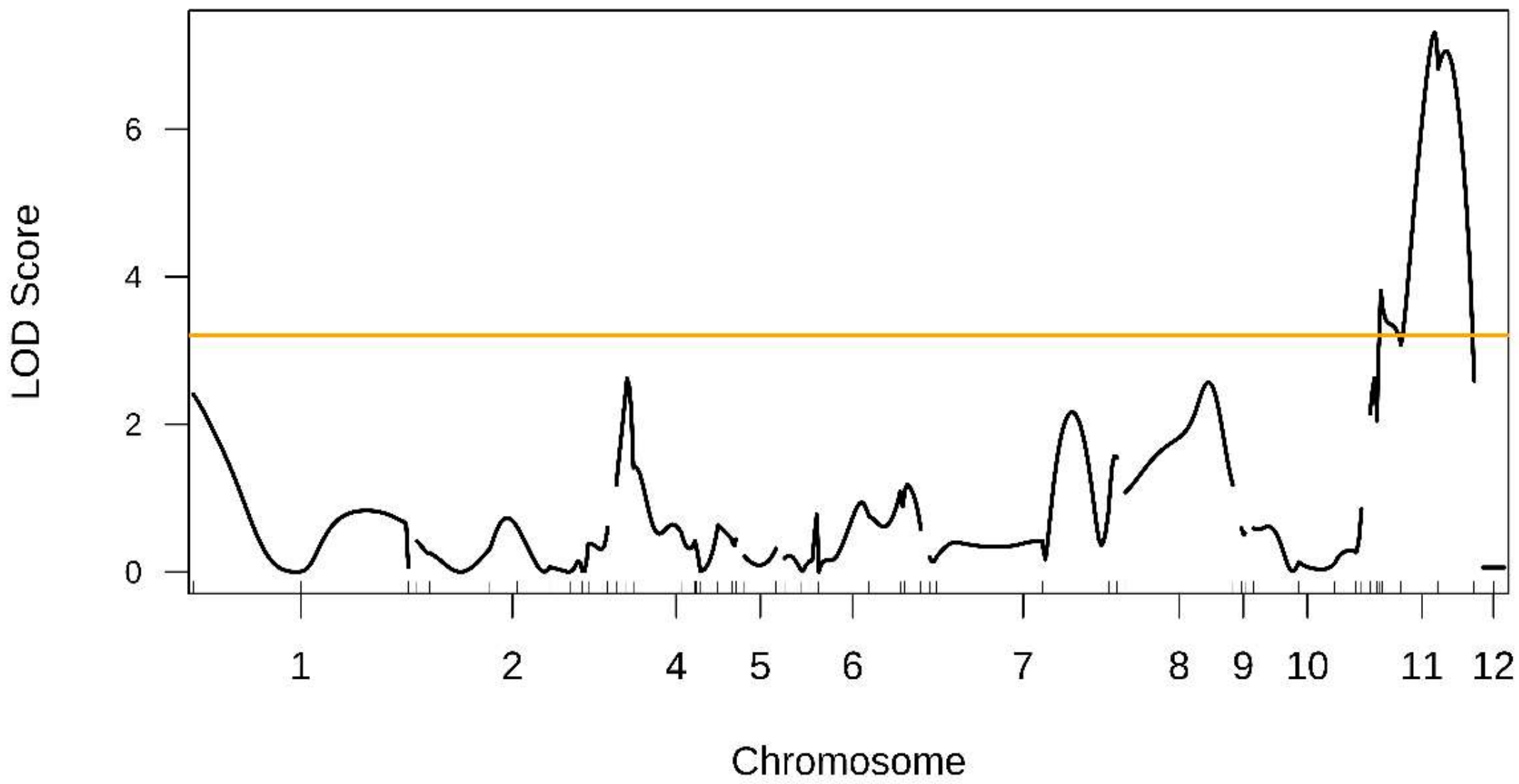

Supplemental Fig. 1 Genome-wide QTL analysis for PRI in the F_5 population. The figure shows LOD scores across 11 rice chromosomes (excluding chromosome 3) based on composite interval mapping using the F_5 population derived from a cross between Ikame1470 and Natsukirari. The y-axis indicates the LOD score, and the x-axis represents marker positions along each chromosome. The orange dashed line represents the genome-wide 5% significance threshold (LOD = 3.28), calculated from 1,000 permutations of the phenotypic data. Chromosome 3 was excluded from the analysis because no polymorphic markers were available in this region. A significant QTL was detected on chromosome 11

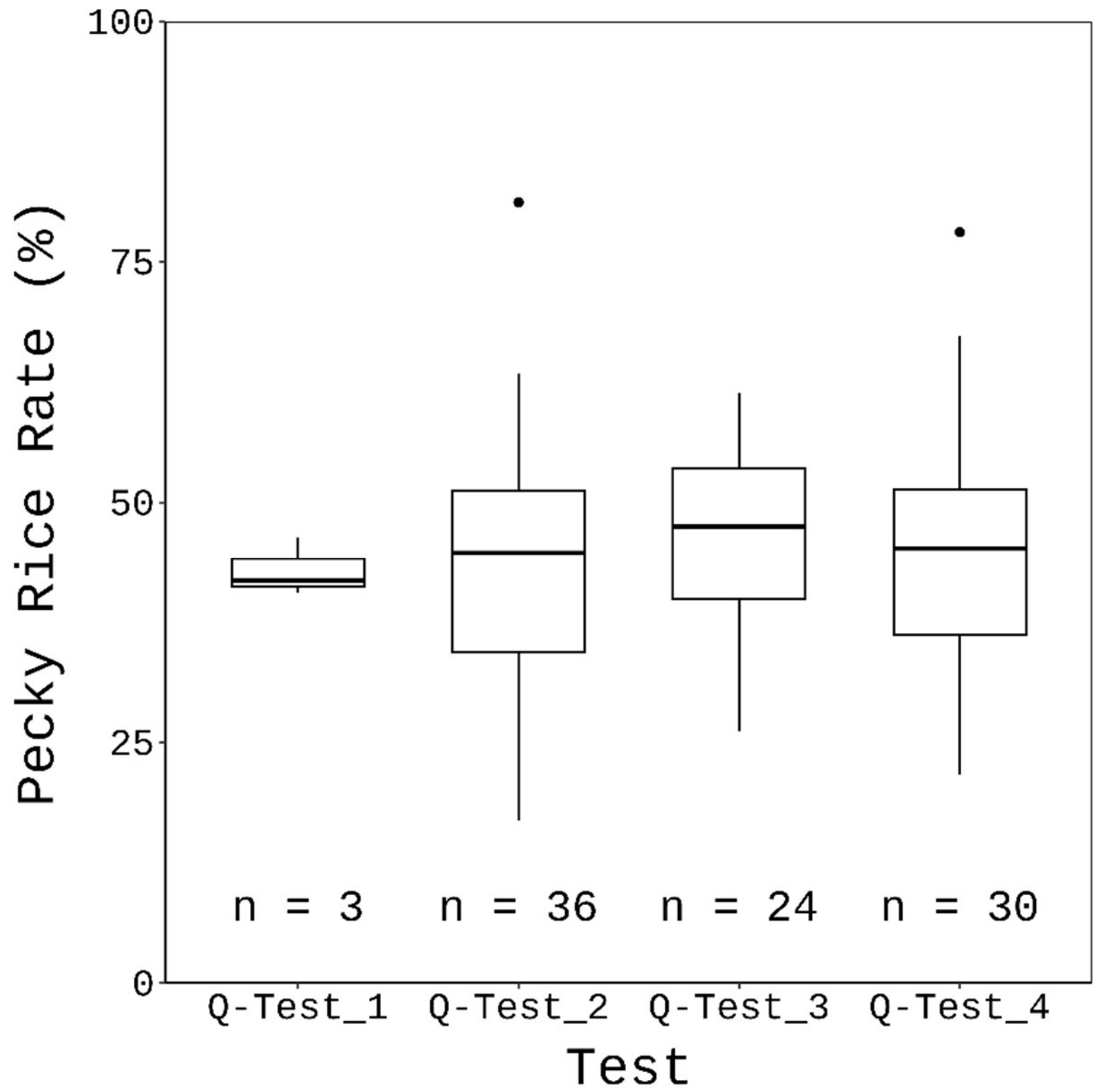

Supplemental Fig. 2 Distribution of raw pecky rice rate across experimental trials in the F_5 population. Box plots show the distribution of pecky rice rate for each of the four independent trials conducted in 2018. The box represents the interquartile range (IQR), with the central line indicating the median. Median values were consistent across trials (coefficient of variation = 5.23%), and no significant differences were detected among trials (Kruskal–Wallis test, $H = 1.14$, $p = 0.77$), indicating stable phenotypic evaluation across experiments.

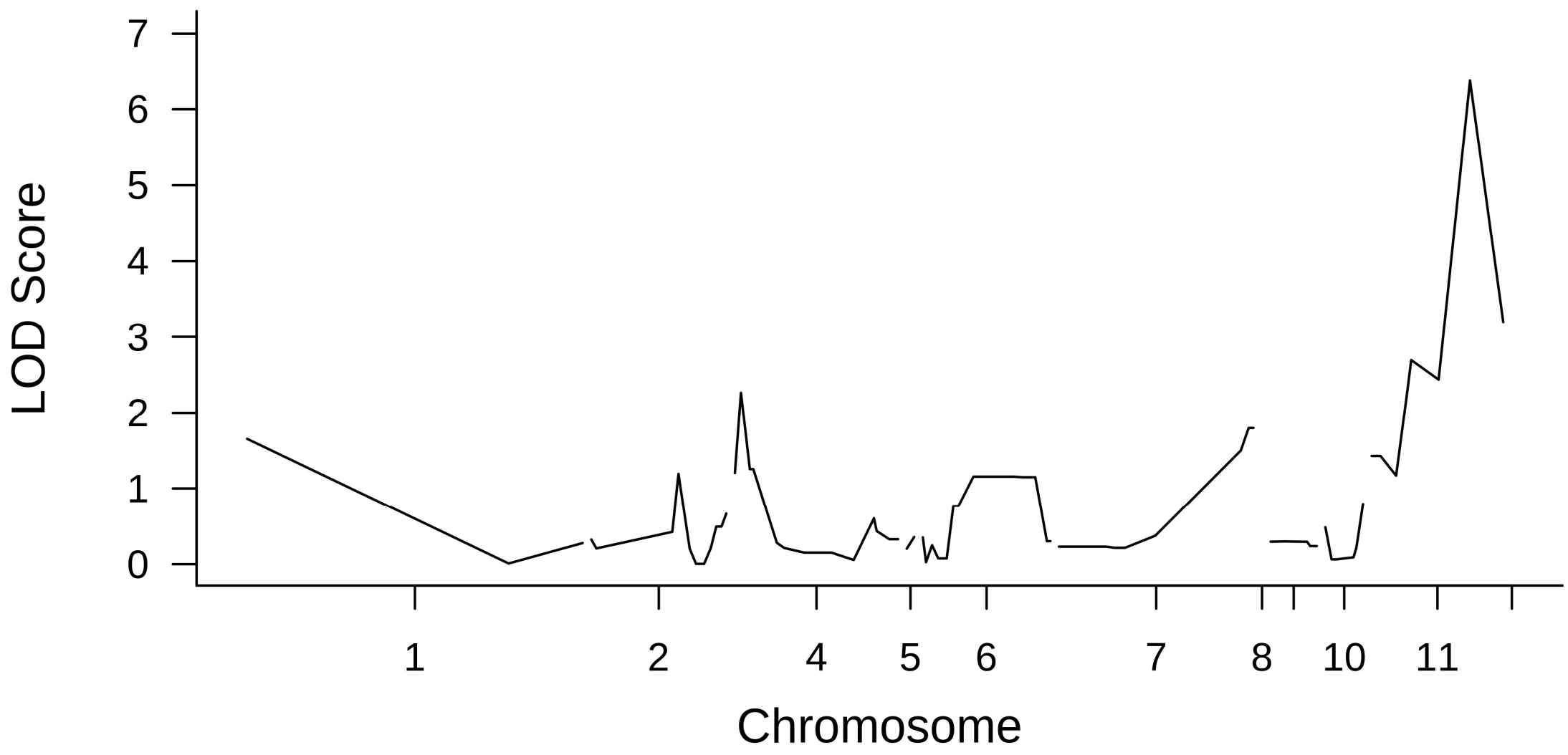

Supplemental Fig. 3 Validation of *qSBR11* using linear model analysis with trial as a covariate. Genome-wide QTL mapping using raw pecky rice rate as the phenotypic value. Linear models included trial as a covariate to account for environmental variation among experimental batches (Model: Pecky rice rate ~ Genotype + Trial).

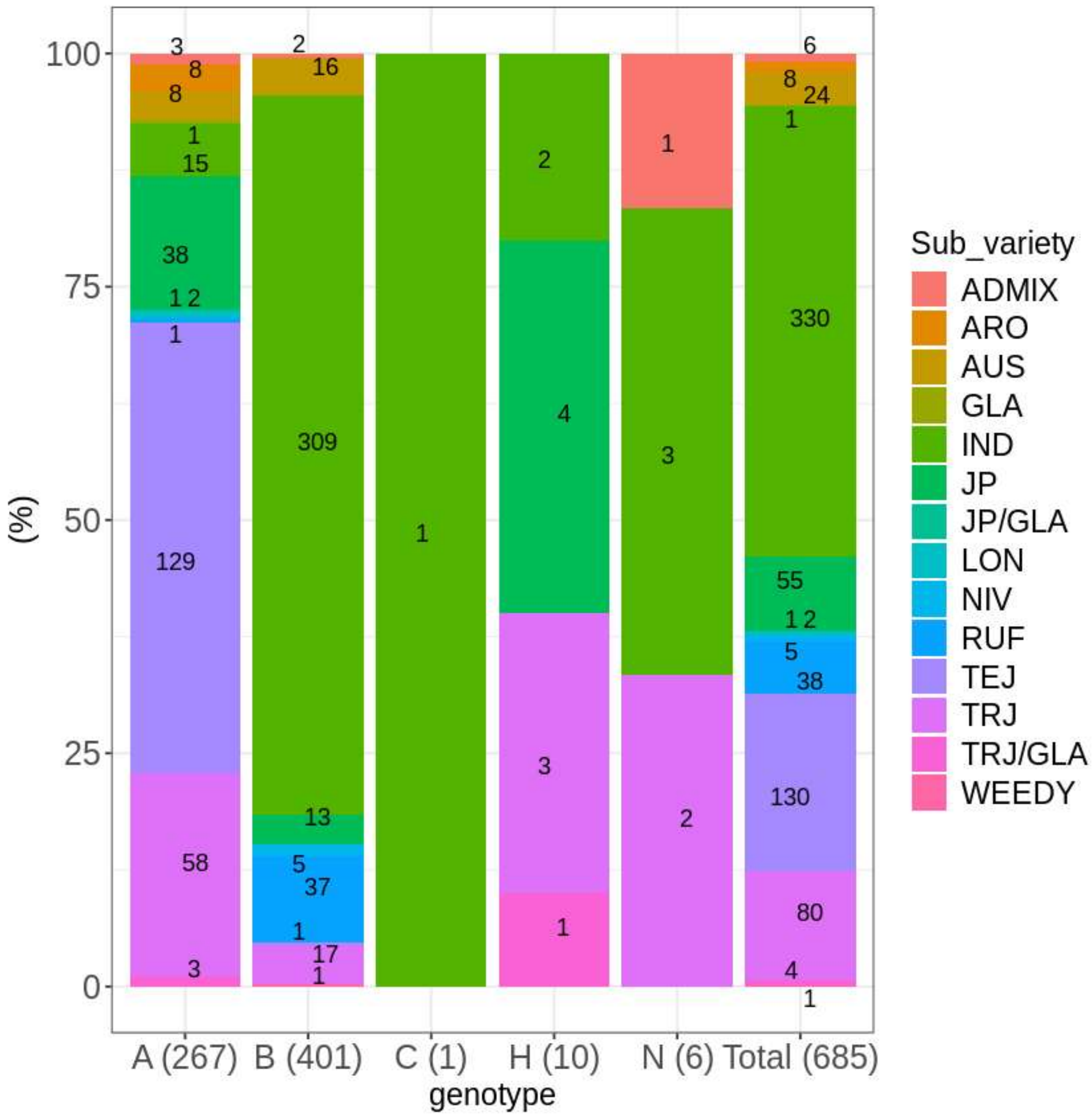

Supplemental Fig. 4 Distribution of C5_indel9205 genotypes across rice subpopulations based on 685 accessions in TASUKE+ for RAP-DB. Genotypic distribution of the C5_indel9205 marker in 685 rice accessions retrieved from the RAP-DB database. The marker genotypes are classified as follows: A = susceptible type (S), B = resistant type (R), C = other allele, H = heterozygous (S/R), and N = missing data. Genotype frequencies are shown as stacked bar graphs by subpopulation. Subpopulation abbreviations: ADMIX = admixture, ARO = aromatic, AUS = *aus*, GLA = *O. glaberrima*, IND = *indica*, JP = *japonica*, JP/GLA = *japonica* / *Glaberrima* mix, LON = long-grain rice, NIV = *O. nivara*, RUF = *O. rufipogon*, TEJ = temperate *japonica*, TRJ = tropical *japonica*, TRJ/GLA = tropical *japonica* / *Glaberrima* mix, WEEDY = weedy *japonica*
