# Supplementary material for: A major quantitative trait locus qSBR11 from Oryza sativa ‘Milyang 44’ confers resistance to rice stink bugs
Source: Breed Sci. 2026 Jun 16;76(3):275–84. doi: 10.1270/jsbbs.25084 (PMC13709644; doi:10.1270/jsbbs.25084)
Supplement: Supplementary file 2 [file 76_275_s2.pdf]

doi: 10.1270/jsbbs.25084

Supplemental Table 1 List of SNP markers used for QTL analysis. Marker ID, chromosome number, genomic position, and parental genotypes are shown

No.	Marker ID	Chr	Position (bp)	Genotype (Natsukirari)	Genotype (Ikame1470)
1	FA0056	1	4,340,906	A	B
2	FA1895	1	34,661,749	A	B
3	FA4804 [a]	1	43,252,861	A	B
4	FA2437	2	19,987,598	A	B
5	FA4826	2	20,577,358	A	B
6	FA1964	2	29,384,660	A	B
7	FA5539 [a]	2	30,097,950	A	B
8	FA4834	2	31,398,112	A	B
9	FA0184	2	32,133,628	A	B
10	FA5874	2	33,074,293	A	B
11	FA2473	2	33,841,459	A	B
12	FA0858	2	34,484,600	A	B
13	FA0188	2	35,089,605	A	B
14	FA4841	2	35,642,862	A	B
15	FA2561	4	297,135	A	B
16	FA2570	4	1,000,941	A	B
17	FA4880	4	2,029,024	A	B
18	FA2583	4	2,432,432	A	B
19	FA2606	4	5,156,085	A	B
20	FA0977	4	6,004,947	A	B
21	FA2624	4	8,324,159	A	B
22	FA2635	4	9,277,769	A	B
23	FA2657	4	11,481,987	A	B
24	FA2675	4	14,074,820	A	B
25	FA1012	4	16,424,235	A	B
26	FA0270	4	16,740,388	A	B
27	FA2705	4	18,196,199	A	B
28	FA2717	4	19,224,907	A	B
29	FA1815	5	27,857,406	A	B
30	FA1127	5	28,726,152	A	B
31	FA1149	6	6,560,336	A	B
32	FA3006	6	6,930,228	A	B
33	FA3949	6	7,631,017	A	B

34	FA4950[a]	6	8,346,062	A	B
35	FA3979[a]	6	8,851,918	A	B
36	FA5499[a]	6	9,333,525	A	B
37	FA4007	6	10,103,190	A	B
38	FA3025	6	10,693,033	A	B
39	FA4953	6	12,426,078	A	B
40	FA3029	6	13,187,837	A	B
41	FA3030	6	14,007,103	A	B
42	FA4956	6	14,385,273	A	B
43	FA1172	6	14,938,560	A	B
44	FA1831	6	15,783,955	A	B
45	FA2088	6	16,417,487	A	B
46	FA5274	6	17,146,779	A	B
47	FA1177	6	18,150,329	A	B
48	FA4960	6	19,150,183	A	B
49	FA2081	6	19,598,290	A	B
50	FA1185	6	20,951,609	A	B
51	FA1186	6	21,351,139	A	B
52	FA4042	7	7,076,561	A	B
53	FA4059	7	7,902,877	A	B
54	FA5525	7	9,152,479	A	B
55	FA3111	7	12,570,358	A	B
56	FA5609	7	13,550,116	A	B
57	FA1257	7	13,992,064	A	B
58	FA4987	7	14,729,387	A	B
59	FA0448	7	18,237,155	A	B
60	FA3155	7	28,167,516	A	B
61	FA1311	7	29,077,790	A	B
62	FA5441	7	29,626,909	A	B
63	FA2160	8	2,079,257	A	B
64	FA4177	8	3,498,803	A	B
65	FA1403	9	420,995	A	B
66	FA3222	9	2,031,461	A	B
67	FA1409	9	3,964,080	A	B
68	FA3225	9	4,641,583	A	B
69	FA3226	9	5,004,736	A	B
70	FA3231	9	5,767,364	A	B

71	FA3313	10	17,598,222	A	B
72	FA3315	10	18,314,783	A	B
73	FA2201	10	18,890,380	A	B
74	FA3323	10	20,858,788	A	B
75	FA1522	10	21,189,435	A	B
76	FA3327	10	21,968,795	A	B
77	FA4604	11	6,542,270	A	B
78	FA3355	11	7,568,617	A	B
79	FA1553	11	9,378,234	A	B
80	FA3359	11	10,700,504	A	B
81	FA2245	11	11,129,975	A	B
82	FA1577	11	14,306,158	A	B
83	FA1597	11	17,945,021	A	B
84	FA1617	11	21,800,375	A	B
85	FA1662[a]	12	1,470	A	B

[a] These six markers were completely fixed across all 94 individuals. The remaining 79 markers showed heterozygous genotypes in at least one individual, indicating residual segregation at the F_5 generation.

Supplemental Table 2 Phenotypic evaluation of the parental lines Ikame1470 and Mi7 for stink bug resistance across multiple field trials. Statistical significance was evaluated using a two-sample t-test. Asterisks indicate significance levels: *$p < 0.05$, **$p < 0.01$, n.s. = not significant. Milyang 44 was included in 2021 as a reference for resistant donor phenotype

Test Date	Pecy Rice Rate (%)						
	Susceptible Control	Ikame 1470		Mi7		Milyang 44	
2015.08.13	65.6	39.3	**	-		-	
2017.08.24	62.3	30.0	*	-		-	
2017.09.13	40.3	15.4	*	-		-	
2020.09.16	57.3	-		27.0	*	-	
2021.09.21	46.6	27.5	n.s.	23.0	*	10.1	**
2022.09.13	40.6	-		25.3	*	-	

Supplemental Table 3 List of DNA markers used in this study. The genomic position represents the 5′ end of the forward primer based on the IRGSP-1.0

Name	Primer sequence (5'–3')	Chr	Position	Amplicon size (bp)		Reference
				R-type	S-type	
C5_indel9182	F: GTGGGCCTTGGTTTGCTT	11	16,329,152	159	142	Yonemaru et al., 2015
	R: TTGCATGATGTAATGTGGTTGA					
MT165166	F: CGGAGGTTGGACCGGCGATGG	11	16,660,414	120	446	This study
	R: TCCTCCCCAATGCCGACGAGC					
MT212213	F: TGGTGAGGGAGAGAGAGGAAAG	11	16,864,768	158	423	This study
	R: TGCACTAAATGCCAAAACCG					
C5_indel9205	F: GTTTATTGTCTTCGGACCTCCA	11	17,069,569	136	119	Yonemaru et al., 2015
	R: GGCACGCATTGAATAAAGTATG					
MT227228	F: CAAGAGCTCTCAACTTCTCACAAC	11	17,480,679	425	109	This study
	R: TCAGTTGCCTCACATTCACTC					
MT099100	F: TTGGTTTCGATCCCTATGG	11	18,062,246	113	140	This study
	R: AAACTTTCGATTCGATCTGG					
C5_indel9234	F: GTCGACGTGTCATACCTTCTGA	11	18,499,644	151	138	Yonemaru et al., 2015
	R: GCAAGATCAAGCTAGCAAGAAA					
RM26810	F: GTTCTTCACCTTCCAGCTACCG	11	18,753,123	170	173	McCouch et al. 2002
	R: CCTACTCCTACTCTTCCCATTTCC					
C5_indel9236	F: TGAAGGTCTACCTGTCATCTTT	11	18,959,128	172	150	Yonemaru et al., 2015
	R: GGTTAGGTTGTAGTTGGTGCAA					
RM5349	F: CATCCAAATGTTGCGGATTACC	11	19,650,332	175	186	McCouch et al. 2002
	R: TTCAATAGCCCAGAGAACCAAGC					

R-type: resistant allele (Milyang 44 type); S-type: susceptible allele (Natsukirari/Aichinokokoro type).

Supplemental Table 4 Genotype profiles of *qSBR11* flanking markers and resistance phenotype in diverse rice cultivars. Genotypes at three markers near *qSBR11* (MT165167, C5_indel9205, and MT227229) are shown for 22 cultivars previously evaluated for stink bug resistance. R = resistant-type allele (Milyang 44 type), S = susceptible-type allele (*japonica* type), H = heterozygous, N = no PCR amplification. Resistance phenotype is based on multi-year field data

		Genotype			
**Cultivar**	**Origin**	**MT165167**	**C5_indel9205**	**MT227229**	**Resistance**
Guangluai 4	China	N	R	R	R
Nanjing 11	China	N	R	R	R
GP242	China	R	R	R	R
Yanxuan 203	China	N	R	R	R
Kenanxuan 10	China	N	R	H	R
GP206	China	R	R	R	R
Ukoku	China Taiwan (landrace)	N	R	R	R
TI-11-8		N	R	R	R
IR 56	IRRI	N	R	S	R
Zhaiyeqing 8	China	N	R	R	R
Yanxuan	China	N	R	R	R
GP201	China	R	R	R	R
Yangdao 1	China	R	R	R	R
Dellmont		S	S	R	R
Shuangfeng 4	China	S	S	S	S
Yangnuo 5	China	S	S	S	S
Nankeng 11	China	S	S	S	S
TIMIS		S	S	S	S
Dian Yu 1		S	S	S	S
Calrose No.2	USA	S	S	S	S
Jakou	Japan (landrace)	S	S	S	S
Henroyori	Japan (landrace)	S	S	S	S
